# Supplementary figures and images for: Listeria monocytogenes Modulates Macrophage Inflammatory Responses to Facilitate Its Intracellular Survival by Manipulating Macrophage-Derived Exosomal ncRNAs
Source: Microorganisms. 2025 Feb 13;13(2):410. doi: 10.3390/microorganisms13020410 (PMC11858176; doi:10.3390/microorganisms13020410)

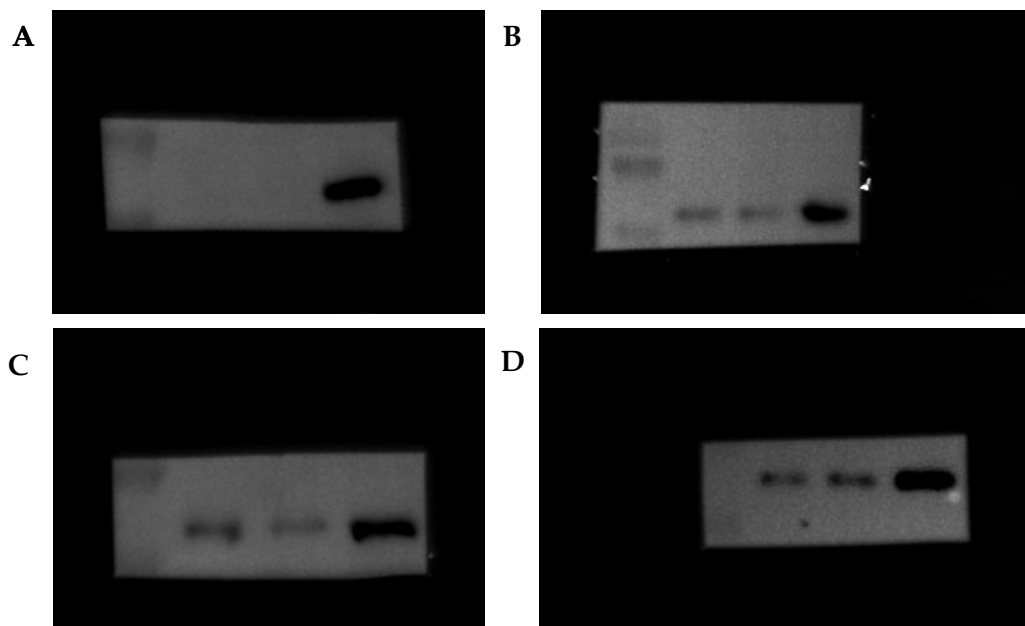

**Figure S1.** uncropped Western Blot. (A)Calnexin (B)CD9 (C)CD63 (D)TSG101

Supplement: Supplementary file 1 [file microorganisms-13-00410-s001.zip › microorganisms-3436487-supplementary.pdf]
